# Suppression of Parathyroid Hormone as a Proxy for Optimal Vitamin D Status: Further Analysis of Two Parallel Studies in Opposite Latitudes

**DOI:** 10.3390/nu12040942

**Published:** 2020-03-28

**Authors:** Marcela M. Mendes, Kathryn H. Hart, Susan A. Lanham-New, Patrícia B. Botelho

**Affiliations:** 1 Department of Nutritional Sciences, University of Surrey, Guildford GU2 7XH, UK; k.hart@surrey.ac.uk (K.H.H.); s.lanham-new@surrey.ac.uk (S.A.L.-N.); 2 Department of Nutrition, Faculty of Health Sciences, University of Brasília, Brasília 70910-900, Brazil; patriciaborges.nutri@gmail.com

**Keywords:** 25-hydroxyvitamin D, optimal levels, parathyroid hormone, vitamin D, Brazil

## Abstract

Optimal vitamin D status has commonly been defined as the level of 25-hydroxyvitamin D (25(OH)D) at which parathyroid hormone (PTH) concentrations would be maximally suppressed, represented by an observed minimum plateau. Previous findings indicate a large variation in this plateau, with values ranging from <30 nmol/L up to 100 nmol/L. This disparity in values might be explained by differences in study design and methodology, ethnicity, age, gender and latitude. This study aimed to investigate the concentration of 25(OH)D at which PTH concentrations were suppressed in Brazilian women living in opposite latitudes (high vs. low: i.e., UK and Brazil), during wintertime. Using data from the D-SOL study (Interaction between Vitamin D Supplementation and Sunlight Exposure in Women Living in Opposite Latitudes), the association between 25(OH)D status and PTH levels were examined in 135 Brazilian women (56 living in England and 79 living in Brazil, aged 20–59 years old). Mean PTH concentrations for Brazilian women with vitamin D deficiency (<25 nmol/L) were significantly higher compared to those with vitamin D insufficiency (25–49.9 nmol/L) (*p* < 0.01), vitamin D adequacy (50–74.9 nmol/L) (*p* < 0.01) and those with optimal vitamin D status (>75 nmol/L) (*p* < 0.001). Regression modelling was used to investigate the relationship between serum 25(OH)D and PTH for the sample as a whole and for each group separately. A cubic model was statistically significant for the total sample (*p* < 0.001), whereas a linear model presented the best fit for Brazilian women living in England (*p* = 0.04) and there were no statistically significant models fitted for Brazilian women living in Brazil. The cubic model suggests that 25(OH)D concentrations above 70–80 nmol/L are optimal to suppress the parathyroid gland in Brazilian women. These findings contribute to a better understanding of the relationship between 25(OH)D and PTH in populations living in a low latitude location and are of great relevance for discussions regarding the estimation of optimal cut-offs for vitamin D levels in the Brazilian population as well as for other low latitude locations.

## 1. Introduction

The major physiological function of vitamin D and its metabolites is to maintain calcium homeostasis for metabolic functioning, signal transduction and neuromuscular activity. The biologically active metabolite 1,25-dihydroxyvitamin D (1α,25(OH)_2_D_3_) is involved in bone formation as well as bone maturation and is the only known hormone to induce the proteins involved in active intestinal calcium absorption [1,2,3]. Along with parathyroid hormone (PTH), it regulates calcium and phosphorous metabolism and enhances the absorption of calcium in the gut and reabsorption of filtered calcium in the kidney [1,2,3,4,5].

When calcium concentrations decrease below normal physiological levels, calcium-sensing proteins stimulate the secretion of PTH and the expression of PTH gene in order to restore calcium homeostasis [1,2,3,4,5]. Consequently, the active 1α,25(OH)_2_D_3_ hormone stimulates intestinal calcium absorption, or along with PTH, in the case of higher concentrations of the latter, increases mobilization of calcium from the bone and renal calcium reabsorption [6,7,8]. Therefore, prolonged elevated PTH concentrations will have a significant negative impact on bone health, consequently increasing the risk of fractures and falls [6,7,8].

Inadequate vitamin D status, as well as inadequate dietary vitamin D and calcium intakes, are considered independent factors linked to increased PTH concentrations [7,9]. Due to the fundamental role of PTH in bone metabolism, the most common criteria used for determining the optimal concentration for serum 25-hydroxyvitamin D (25(OH)D, the circulating metabolite) for bone health in adults, include the suppression of PTH secretion [9,10,11,12,13]. Other parameters that may also be used for determining optimal vitamin D status include higher bone mineral density, along with reduced rates of bone loss and decreases in fractures and falls [9,10,11,12,13]. 

The relationship between PTH and vitamin D serum concentrations has been largely demonstrated so far, however most studies were conducted in elderly and/or osteoporotic populations [9,10,12,13,14], and moreover in high latitude countries (defined as latitude greater than 40° N or S), such as the United States, Canada and most of Europe, including the United Kingdom. It is important to note that in adults, increases in PTH may not be determined the same way and may not be as detrimental to bone health as it has been shown to be in older populations. Moreover, vitamin D status can have an independent protective effect on bone that would attenuate the negative effect of high PTH levels. Thus, there is still a significant gap in our knowledge as to the importance of PTH suppression and a considerable lack of data on healthy younger adults, particularly in low latitudes countries (lower than 40° N or S), such as the African continent, Australia and most of Latin America, including Brazil, where there is a higher and more constant level of sun exposure all year round [15,16].

Specific to the population groups living in low latitude locations, very few consistent studies so far have evaluated vitamin D status in relationship with PTH. Thus, further data collection is needed to better determine specific vitamin D recommendations for this particular papulation group and geographical location. This study, therefore, aimed to determine the relationship between serum 25(OH)D and PTH concentrations and to investigate whether there is a threshold for serum 25(OH)D concentrations where a minimum plateau in PTH is evident in Brazilian women living in opposite latitudes.

## 2. Materials and Methods

This is a cross-sectional analysis of 25(OH)D and PTH concentrations, calcium and vitamin D intake, conducted in 135 healthy adult (aged 20 to 59 years) Brazilian (born in Brazil and having at least one parent born in Brazil) women, living in either England (51° N) or Brazil (16° S), recruited for the D-SOL study (Interaction between Vitamin D Supplementation and Sunlight Exposure in Women Living in Opposite Latitudes—clinicaltrials.gov as NCT03318029). 

The study received a favorable ethical opinion from the University of Surrey (UEC/2016/009/FHMS) and the Federal University of Goiás Ethics Committees and from the Brazilian National Ethics Committee (CONEP) (CAAE/62149516.9.0000.5083, CEP-UFG/2013222; CONPEP/1972029; respectively).

### 2.1. Exclusion Criteria

The exclusion criteria included potential cofounders likely to affect vitamin D metabolism (osteoporosis therapy, anti-estrogen treatment, antiepileptic drugs and cancer treatment), taking supplements containing vitamin D (if the prospective participants agreed to stop vitamin D supplementation to join the study, a wash-out period of 8 weeks prior to commencing the trial was accepted), being pregnant or planning a pregnancy during the study period, menopause status (defined as the permanent cessation of menstruation) and living in the UK for less than 3 months at the commencement of the study (for England participants only). All participants provided written informed consent at the commencement of the study.

### 2.2. Data Collection

The dietary intake of vitamin D and calcium was determined by 4 consecutive days of estimated diet diaries, including one weekend day. Participants were instructed by the research team on how to correctly complete the diary and asked to give as much detail as possible of every meal, including portion size.

Dietary intake data obtained from participants in the England cohort were analyzed using the Nutritics^®^ nutritional analysis software, version 4.0, Dublin, Ireland (UK Composition of Foods Integrated Dataset (CoFID) including McCance and Widdowson 7th edition [17]) and those collected in Brazil were analyzed via the Dietwin^®^ software Version 13 (3090), Rio Grande do Sul, Brazil (Brazilian Food Composition Table (TACO) [18] and the food composition database from the Brazilian Institute of Geography and Statistics (IBGE), as well as the United States Department of Agriculture (USDA) food composition database).

An overnight fasted (8 h) blood sample was collected by venipuncture by trained phlebotomists. Processed serum and plasma samples were divided into aliquots and stored at −80 °C at the University of Surrey, prior to analysis. Samples collected in Brazil followed the exact same procedures and were temporarily stored at −80 °C at the Federal University of Goiás and sent to be stored at the University of Surrey as well, prior to analysis.

### 2.3. Laboratory Analysis

Serum 25(OH)D concentrations were determined by the HPLC-MS/MS method on a Waters Acuity UPLC (Triple Quadrupole) TQD^®^ System using a Pentafluorophenyl (PFP) column following supported liquid extraction (SLE). Laboratory intra- and inter-assay coefficients of variation (CV) were 5.6% and 7.8%, respectively. Calcium, albumin and PTH concentrations were measured using Abbott Architect kits. Serum calcium was measured by using an endpoint spectrophotometric reaction based on the o-cresolphthalein complexone methodology, and serum albumin was measured by using an endpoint spectrophotometric reaction based on the bromocresol green solution dye binding methodology. Serum calcium concentrations were adjusted for albumin concentrations. Plasma intact PTH was measured by in vitro chemiluminescent microparticle immunoassay (CMIA). The manufacturer’s quoted inter-assay CV for calcium was <3%, for albumin <3.8% and for PTH 4%.

The definition of optimal 25(OH)D status is still very debated within the scientific community and continues to be controversial amongst different recommendations. For the purpose of this study, vitamin D deficiency was defined as 25(OH)D concentrations below 25 nmol/L, as suggested by the UK Scientific Advisory Committee on Nutrition [19]; insufficiency as concentrations between 25–49.9 nmol/L and adequacy between 50–74.9 nmol/L, as recommended by the US Institute of Medicine [20]; and optimal levels as concentrations above 75 nmol/L, as proposed by the US Endocrine Society [12]. 

### 2.4. Statistical Analysis

Statistical analysis of the data was undertaken using SPSS software for Windows (version 25.0, IBM Corp, Armonk, NY, USA). Data were tested for normal distribution using the Kolmogorov–Smirnov tests. Non-normally distributed variables were log transformed and reported on the original scale. Non-parametric tests were used when log transforming did not normalize the data. Descriptive statistics were determined for all variables. Continuous variables are presented as mean ± standard deviation (SD). Baseline characteristics (age, dietary intakes and biomarkers) were compared between countries, by independent t-tests or Mann–Whitney. 

For the whole sample and in each country separately, Pearson (or Spearman) correlation tests were used to investigate the relationship between PTH concentrations and serum 25(OH)D, calcium concentrations and vitamin D and calcium intakes. 

One-way ANOVA with post-hoc Tukey tests were used to compared PTH concentration between different vitamin D statuses. One-way ANOVA and regression model fit tests were conducted for both original and normalized PTH data (log transforming) for all analysis and there were no differences in the statistical outcome, therefore only analysis with original data is reported.

Regression modelling was used to model the relationship between serum 25(OH)D and PTH concentrations for the whole sample and in each country separately. A linear model represents no plateau in the analyzed data whereas cubic or quadratic models suggest a plateauing pattern.

A p value of <0.05 was considered significant. For comparisons between groups, effect size based on eta squared was defined as 0.01 as a small effect, 0.06 as a medium effect and 0.14 as a large effect.

## 3. Results

**Figure 1:**
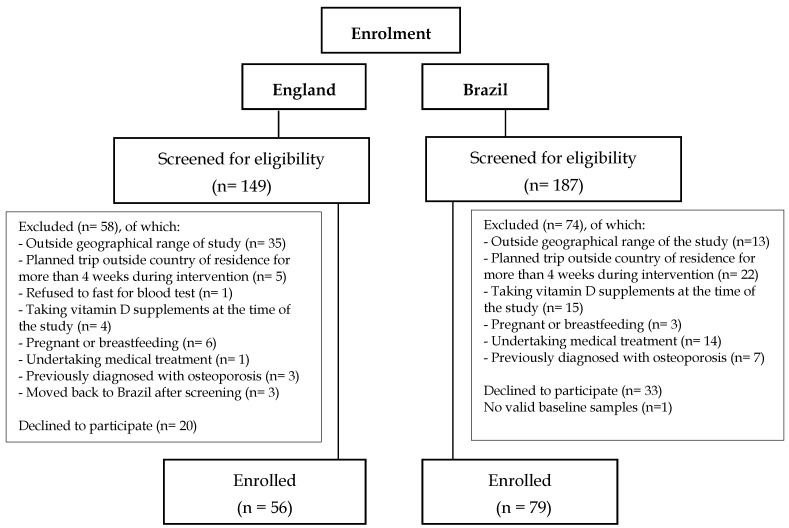
Flow diagram of participant enrolment.

A total of 336 adult Brazilian women were screened for the study, 149 in England and 187 in Brazil, of which 132 were excluded based on the exclusion criteria, 53 decided not to participate after the screening process and 15 did not attend their baseline visit. Reasons for exclusion at screening are detailed in Figure 1. In the Brazil cohort one participant did not have valid laboratory results and was therefore excluded from database (Figure 1).

Of the participants enrolled for the D-SOL study after the screening process, 135 participants were included in this cross-sectional analysis (*n* = 56 in England and 79 in Brazil) (Figure 1). In the England cohort five participants were post-menopausal. There were no differences between analyses including or excluding the 5 post-menopausal women, and therefore, only analyses including these participants are reported here.

### 3.1. Participant Characteristics

Participants characteristics for age, weight, BMI, vitamin D and calcium intake and 25(OH)D, PTH and calcium serum concentrations have been previously published in Mendes et al., 2019 [21]. Brazilian women living in England were older, heavier and had a greater waist circumference than those living in Brazil (*p* < 0.01). There were no significant differences between Brazilian women living in England and in Brazil for BMI classification distributions although, in line with the weight data, the mean BMI was significantly greater for Brazilian women residing in England.

Overall (*n* = 135) mean habitual vitamin D dietary intake was 2.4 ± 1.9 μg/day and mean calcium intake was 625.1 ± 310.7 mg/day. Mean vitamin D and calcium intakes were significantly higher for women living in England compared to women living in Brazil (*p* = 0.012 and *p* = 0.001, respectively) (Table 1). 

In participants living in England, mean 25(OH)D concentration was significantly lower than for participants in Brazil (*p* < 0.001) (Table 1). There were no significant differences between Brazilian women living in England and Brazilian women living in Brazil for serum calcium concentrations (*p* = 0.066), which were within the normal range for all participants (Table 1).

### 3.2. Plasma PTH Concentrations

Mean PTH concentration in Brazilian women living in England was significantly higher than within Brazilian women living in Brazil (5.36 ± 1.99 pmol/L and 4.49 ± 1.47 pmol/L, respectively *p* = 0.004) (Table 1). PTH concentrations ranged from 2.41 to 13.76 pmol/L within those living in England and from 2.18 to 8.51 pmol/L in participants living in Brazil.

PTH concentrations were negatively correlated with 25(OH)D concentrations (*r* = −0.285, *p* = 0.001) and there was no significant correlation between PTH and calcium concentrations (*r* = −0.093, *p* = 0.285) (data previously published) [20]. There were no statistically significant correlations between PTH concentrations and vitamin D (*r* = 0.083, *p* = 0.372) and or calcium (*r* = 0.049, *p* = 0.596) intakes, even when controlled for 25(OH)D concentrations (all *p* > 0.05). 

### 3.3. Differences in Mean PTH Concentrations by Vitamin D Status

**Figure 2:**
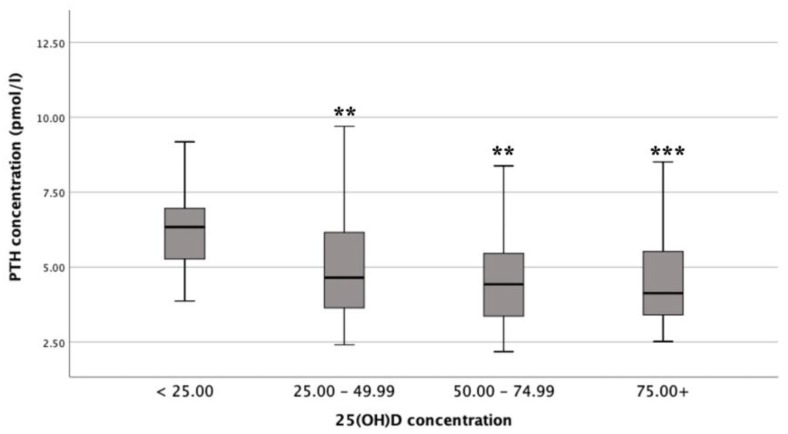
Differences in mean parathyroid hormone (PTH) concentrations by different vitamin D status (*n* = 135). ** *p* < 0.01; *** *p* < 0.001 (ANOVA compared to [25(OH)D] < 25 nmol/L).

There was a statistically significant difference in mean PTH concentrations between the different vitamin D status groups (F (3, 130) = 6.5, *p* <0.001; *n* = 135), with a large actual difference in mean PTH concentrations (effect size = 0.14). Post-hoc comparisons showed that mean PTH concentrations for deficient (6.60 ± 2.47 pmol/L) were significantly different from insufficient (4.96 ± 1.70 pmol/L; *p* = 0.009), adequate (4.55 ± 1.39 pmol/L; *p* = 0.001) and optimal (4.42 ± 1.51 pmol/L; *p* <0.001) status (Figure 2) [12,19,20]. 

### 3.4. 25(OH)D Status at which PTH Concentrations Reach a Minimum Plateau

**Figure 3:**
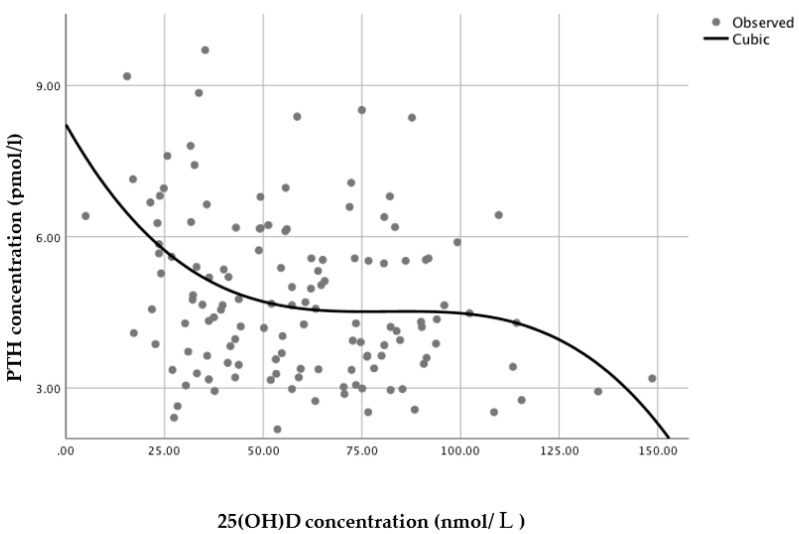
Relationship between serum 25(OH)D and PTH concentrations (*n* = 134, *R*^2^ = 0.113, *p* = 0.001).

A series of regression analyses were conducted to model the relationship between serum 25(OH)D and PTH. For the sample as a whole (*n* = 135), a non-linear regression cubic model was found to have the best statistically significant fit (*n* = 135, *R*^2^ = 0.109, *p* = 0.002), with PTH reaching a minimum plateau at 25(OH)D concentrations of around 70–80 nmol/L, based on the inflection point of the curve (i.e., the curve starts changing from being convex (concave upward) to concave (concave downward) (Figure 3)).

**Figure 4:**
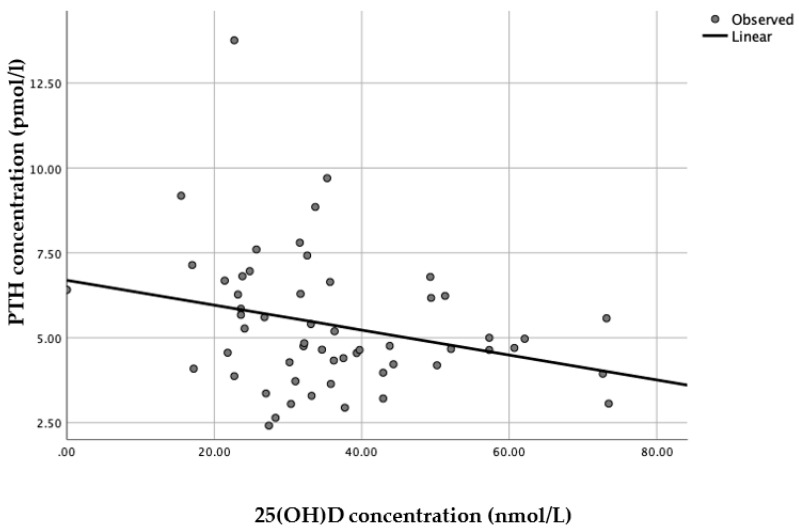
Relationship between serum 25(OH)D and PTH concentrations for Brazilian women living in England (*n* = 56, *R*^2^ = 0.076, *p* = 0.04).

When each latitude was assessed separately, a linear model was presented as the best fit with statistical significance (*n* = 56, R^2^ = 0.076, *p* = 0.04) for Brazilian women living in England (Figure 4), whereas for Brazilian women living in Brazil there was no statistical significance for any of the models applied (*n* = 79, all *p* > 0.25).

### 3.5. Assessment of Higher 25(OH)D, PTH and Calcium Concentrations and Adverse Events

Only Brazilian women living in Brazil had serum 25(OH)D concentrations above 100 nmol/L (*n* = 3), of which two women had concentrations above 130 nmol/L (134.9 and 148.6 nmol/L). Nine Brazilian participants living in England and five living in Brazil had PTH concentrations above 6.9 pmol/L. Serum calcium concentrations were within the reference range for all participants (<2.5 mmol/L). None of the participants reported any adverse events throughout the duration of the study.

## 4. Discussion

Mean PTH concentration in Brazilian women residing in England was significantly higher than within Brazilian women in Brazil, reflecting the significantly lower 25(OH)D concentrations in England dwelling women observed in this study. The observed differences in 25(OH)D concentrations are most likely due to expected differences in sunlight availability and exposure between the two countries. Although there was a significant difference in mean age between the two groups, this would not be likely to influence the state of vitamin D in these two populations since they were relatively young and vitamin D production has been shown to start to decline later in life (over 50–60 years) [22,23]. PTH concentrations were negatively correlated with 25(OH)D concentrations and PTH clearly reached a minimum plateau, representing the suppression of PTH secretion, at 25(OH)D concentrations of 70–80 nmol/L.

The definition of optimal status has been commonly derived from the level of 25(OH)D at which PTH levels reach a minimum plateau, as a surrogate of optimal bone health, but this yields a wide range of estimates. There is, however, a growing consensus that serum 25(OH)D concentrations of at least 75–80 nmol/L are required for optimal bone health [10,24]. The findings of the current analysis are in accordance with this consensus, by suggesting that 25(OH)D concentrations above 70–80 nmol/L would be optimal to reduce stimulation of the parathyroid gland in Brazilian women. These findings are, therefore, of great relevance for discussions regarding the estimation of optimal cut-offs for vitamin D levels in the Brazilian population as well as for other low latitude locations.

Several studies have associated increased PTH concentrations with adverse health outcomes, particularly musculoskeletal issues [10,25,26,27], since secondary hyperparathyroidism is associated with increased bone turnover rates and consequently increased bone loss [25,28]. Several studies have also reported that the serum 25(OH)D concentrations at which PTH levels are suppressed vary substantially, from either as low as 30 nmol/L to up to more than 100 nmol/L, while some other studies found no relation between 25(OH)D and PTH, or found a linear relation without a plateau, meaning no clear suppression of PTH secretion by 25(OH)D [10]. As has been demonstrated in other studies [10,25,27,28], 25(OH)D was shown to be negatively correlated with PTH in the present sample of adult Brazilian women. In this sample, PTH concentrations for vitamin D deficient status were significantly higher compared to the insufficient, sufficient and optimal status [12,19,20]. When analyzed separately a point of inflection of plasma PTH could not be identified for either group, for Brazilian women living in England a linear model was the only statistically significant model whereas within Brazilian women living in Brazil there were no statistically significant models that fitted the data. This could be due to the narrow range of serum 25(OH)D in Brazilian women living in England leading to a linear model and due to the Brazil-dwelling group having higher 25(OH)D concentrations resulting in a less present impact on PTH levels. A study with 389,004 Brazilian individuals of both genders aged 2 to 95 years, throughout the four seasons, reported a sinusoidal interrelationship relationship between 25(OH)D and PTH among individuals living in a subtropical area in Brazil, in which PTH levels showed an inverted pattern of peaks in comparison to 25(OH)D levels [28].

There is a clear need for consistent data to establish realistic and meaningful recommendations of vitamin D requirements, particularly for different ethnic/racial groups. Adding to the challenge on the literature being still very controversial on recommended vitamin D status, currently most recommendations have been based on studies conducted in high latitude countries with older Caucasian populations.

The strengths of the present study include the inclusion of healthy adult Brazilian women, aged 20 to 59 years old, consisting of a representative sample, and the measurement of serum 25(OH)D measurement via liquid chromatography–mass spectrometry. It is important to note that these findings may not be generalizable to other ethnic groups with different characteristics, habits or culture.

When translating this into public health recommendations, the data presented here suggests that the current recommendation for Brazilian adult women of 25(OH)D concentrations above 50 nmol/L [29] might not be sufficient to reduce stimulation of the parathyroid gland in this population, which could lead to serious detrimental effects to bone health, particularly when these women reach menopause. This study shows that concentrations above 70–80 nmol/L would instead be required to better maintain bone health in these women, as observed by the applied cubic model.

## 5. Conclusions

We concur with the current consensus as to the 25(OH)D optimal levels at which PTH secretion is suppressed but demonstrate this in a previously unstudied population, specifically, healthy adult Brazilian women. This data could also contribute to a better understanding of the relationship between 25(OH)D and PTH in populations living in a low latitude location. Moreover, with different generalized guidelines proposing relatively differing definitions of vitamin D status, clinical recommendations on vitamin D dietary intake and need for supplementation might be challenging. Thus, these findings could be of great relevance for further discussions regarding the estimation of optimal cut-offs for vitamin D levels specific for the Brazilian population. Further work to extend the findings in this current research with larger and more diverse sample sizes, including men, is now warranted.

## Figures and Tables

**Table 1:** Characteristics of adult Brazilian women by country of residence (*n* = 135) ^1^.

	England (*n* = 51)	Brazil (*n* = 79)	*p* Value ^2^
Age (years)	35.55 ± 9.03	28.43 ± 7.14	<0.001 ^b^
Vitamin D intake (μg/day)	2.96 ± 1.87	2.08 ± 1.86	0.012 ^b^
Calcium intake (mg/day)	728.49 ± 318.75	547.47 ± 282.79	0.001
Serum 25(OH)D (nmol/L)	36.06 ± 14.97	75.00 ± 22.13	<0.001
Plasma PTH (pmol/L)	5.36 ± 1.99	4.49 ± 1.47	0.004 ^b^
Serum Calcium (mmol/L) ^3^	2.30 ± 0.09	2.28 ± 0.07	0.066 ^b^

^1^ Values are mean ± SD. ^2^ Statistical analysis: Independent *t*-test, unless otherwise stated: ^b^ Mann–Whitney. ^3^ Albumin corrected serum calcium concentrations.
